# SALL4 promotes gastric cancer progression through activating CD44 expression

**DOI:** 10.1038/oncsis.2016.69

**Published:** 2016-11-07

**Authors:** X Yuan, X Zhang, W Zhang, W Liang, P Zhang, H Shi, B Zhang, M Shao, Y Yan, H Qian, W Xu

**Affiliations:** 1 Jiangsu Key Laboratory of Medical Science and Laboratory Medicine, School of Medicine, Jiangsu University, Jiangsu, China

## Abstract

The stem cell factor SALL4 (Sal-like protein 4) plays important roles in the development and progression of cancer. SALL4 is critically involved in tumour growth, metastasis and therapy resistance. However, the underlying mechanisms responsible for the oncogenic roles of SALL4 have not been well characterized. In this study, we demonstrated that SALL4 knockdown by short hairpin RNA greatly inhibited the proliferation, migration and invasion of gastric cancer cells. We further confirmed the inhibitory effects of SALL4 knockdown on gastric cancer cells by using a tetracycline-inducible system. Mechanistically, SALL4 knockdown downregulated the expression of CD44. The results of luciferase assay and chromatin immunoprecipitation study showed that SALL4 bound to CD44 promoter region and transcriptionally activated CD44. The results of rescue study revealed that CD44 overexpression antagonized SALL4 knockdown-mediated inhibition of gastric cancer cell proliferation, migration, and invasion *in vitro* and gastric cancer growth *in vivo*. Collectively, our findings indicate that SALL4 promotes gastric cancer progression through directly activating CD44 expression, which suggests a novel mechanism for the oncogenic roles of SALL4 in gastric cancer and represents a new target for gastric cancer therapy.

## Introduction

Cancer cells share common gene expression signature with stem cells. The studies on the key genes in the maintenance of stem cells have led to the identification of factors that are responsible for the malignant phenotype of cancer cells. SALL4 is a zinc finger transcription factor that governs the self-renewal and pluripotency of embryonic stem cells through the constitution of a core transcriptional circuit with Oct4, Nanog and Sox2.^1, 2, 3^ SALL4 is also suggested as a key regulator in normal hematopoiesis and liver development. SALL4 is expressed in hematopoietic and hepatic stem/progenitor cells and is downregulated after hematopoietic and hepatic differentiation.^4, 5^ In addition, SALL4 is critical for DNA damage response in embryonic stem cells.^6^ SALL4 expression gradually decreases during development and is even absent in most adult tissues. However, there is increasing evidence showing that SALL4 expression is re-stored in cancer.

SALL4 is first found to be aberrantly expressed in human acute myeloid leukemia (AML) and SALL4 transgenic mice have been shown to develop AML.^7^ SALL4 is suggested as a key regulator of leukemic cell survival and SALL4 downregulation leads to significant cell apoptosis.^8^ The roles of SALL4 in AML are associated with the regulation of β-catenin by protein–protein interaction,^7^ the upregulation of Bmi-1 and HOXA9 by transcriptional activation,^7, 9, 10^ and the inhibition of PTEN expression by epigenetic silencing.^11^ SALL4 is also involved in the maintenance of leukemic stem cells through the regulation of ABCA3.^12^

In addition to hematopoietic malignancies, SALL4 is also found to be upregulated in solid tumours including liver cancer,^13, 14^ colon cancer,^15, 16^ breast cancer,^17, 18^ endometrial cancer,^19, 20^ lung cancer^21, 22^ and glioma.^23^ The recent studies also suggest an oncogenic role of SALL4 in solid tumours. In breast cancer, SALL4 could upregulate the expression of ZEB1 to promote breast cancer cell migration and invasion through the induction of epithelial-mesenchymal transition (EMT).^24^ In endometrial cancer, SALL4 promotes cell proliferation, migration, invasion and drug resistance via the upregulation of c-Myc.^19^ The potent roles of SALL4 in cancer has made it a novel biomarker for cancer diagnosis and treatment.^25^ The elevated expression of SALL4 is associated with the malignant phenotype of cancer cells and the adverse prognosis in cancer patients.^26^ Moreover, targeted depletion of the expression and the oncogenic activity of SALL4 have shown promising therapeutic effects in the experimental animal models of AML and liver cancer.^27, 28^ Therefore, better understanding of the mechanisms responsible for the roles of SALL4 in cancer would help facilitate cancer diagnosis and prognosis as well as provide new avenues for anti-cancer therapy.

In our previous study, we have shown that SALL4 is highly expressed in the tumour tissues of human gastric cancer patients and the elevated expression of SALL4 predicts poor prognosis.^29^ However, the underlying molecular mechanisms have not been well understood. In this study, we reported that SALL4 bound to the promoter region of CD44 gene and transcriptionally activated CD44 expression. Stable or inducible knockdown of SALL4 suppressed gastric cancer cell proliferation, migration and invasion, while CD44 overexpression antagonized these inhibitory effects. Our findings indicate that SALL4 promotes gastric cancer growth and metastasis through the activation of CD44, which represents a new mechanism responsible for the oncogenic roles of SALL4 in cancer.

## Results

### Stable knockdown of SALL4 inhibits the proliferation, migration and invasion of gastric cancer cells

**Figure 1:**
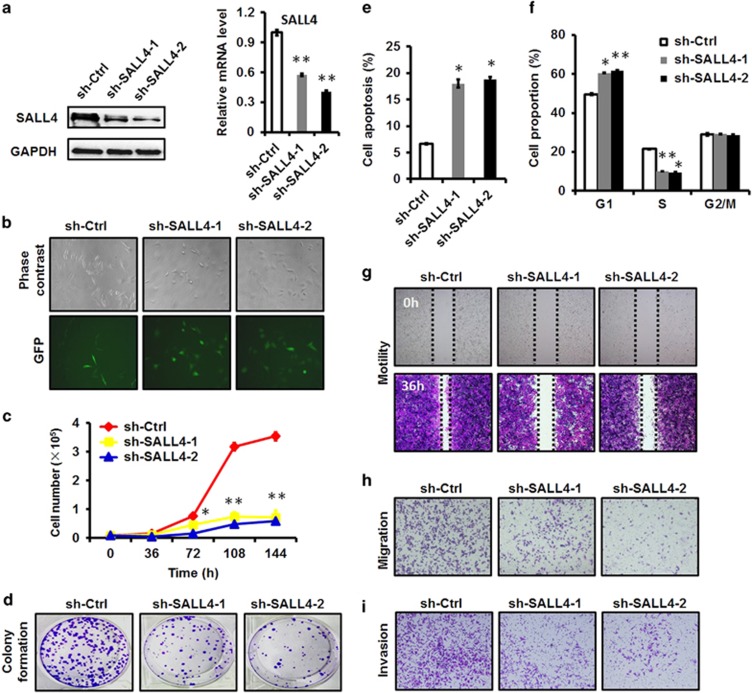
Stable knockdown of SALL4 by shRNA inhibits the proliferation, migration and invasion of gastric cancer cells. (**a**) Quantitative RT-PCR and western blot analyses of SALL4 expression in control and SALL4 knockdown gastric cancer cells. (**b**) The morphology of control and SALL4 knockdown gastric cancer cells. (**c**) Cell counting assay for control and SALL4 knockdown gastric cancer cells. (**d**) Cell colony formation assay for control and SALL4 knockdown gastric cancer cells. (**e**) The percentage of apoptotic cells in control and SALL4 knockdown gastric cancer cells was detected by using Annexin V/PI double staining followed by flow cytometric analysis. (**f**) Cell cycle distribution in control and SALL4 knockdown gastric cancer cells was detected by using PI staining followed by flow cytometric analysis. (**g**) Wound healing assay for control and SALL4 knockdown gastric cancer cells. (**h**) Transwell migration assay for control and SALL4 knockdown gastric cancer cells. (**i**) Matrigel invasion assay for control and SALL4 knockdown gastric cancer cells. *P<0.05, **P<0.01, compared to sh-Ctrl group.

To demonstrate the biological roles of SALL4 in gastric cancer, we suppressed SALL4 expression in gastric cancer cells by using two short hairpin RNAs (shRNAs) that specifically target SALL4. The knockdown efficacy of shRNAs in SALL4 gene and protein expression was verified by using quantitative RT-PCR and western blot (Figure 1a). The knockdown of SALL4 gene by shRNAs downregulated the expression of both SALL4A and SALL4B isoforms (Supplementary Figure 1). We observed significant changes in cell morphology in SALL4 knockdown cells after cell replating (Figure 1b). We then checked the effects of SALL4 knockdown on the malignant phenotypes of gastric cancer cells. We examined the role of SALL4 knockdown in gastric cancer cell growth by using cell counting assay. As shown in Figure 1c, SALL4 knockdown greatly retarded the growth of gastric cancer cells. We further confirmed the inhibitory effect of SALL4 knockdown on the growth of gastric cancer cells by using cell colony formation assay (Figure 1d). We found that SALL4 knockdown induced apoptosis and cell cycle arrest at G1 phase in gastric cancer cells (Figures 1e and f). We next detected the effects of SALL4 knockdown on the motility of gastric cancer cells. Compared to the control cells, SALL4 knockdown cells showed a greatly reduced motility ability (Figure 1g). Finally, we determined the effects of SALL4 knockdown on cell migration and invasion. As shown in Figures 1h and i, SALL4 knockdown greatly reduced the migration and invasion abilities of gastric cancer cells. We also observed that SALL4 knockdown sensitized gastric cancer cells to cisplatin treatment (Supplementary Figure 2). Taken together, these results indicate that stable knockdown of SALL4 by shRNA inhibits the proliferation, migration and invasion of gastric cancer cells.

### Inducible knockdown of SALL4 inhibits the proliferation and migration of gastric cancer cells.

**Figure 2:**
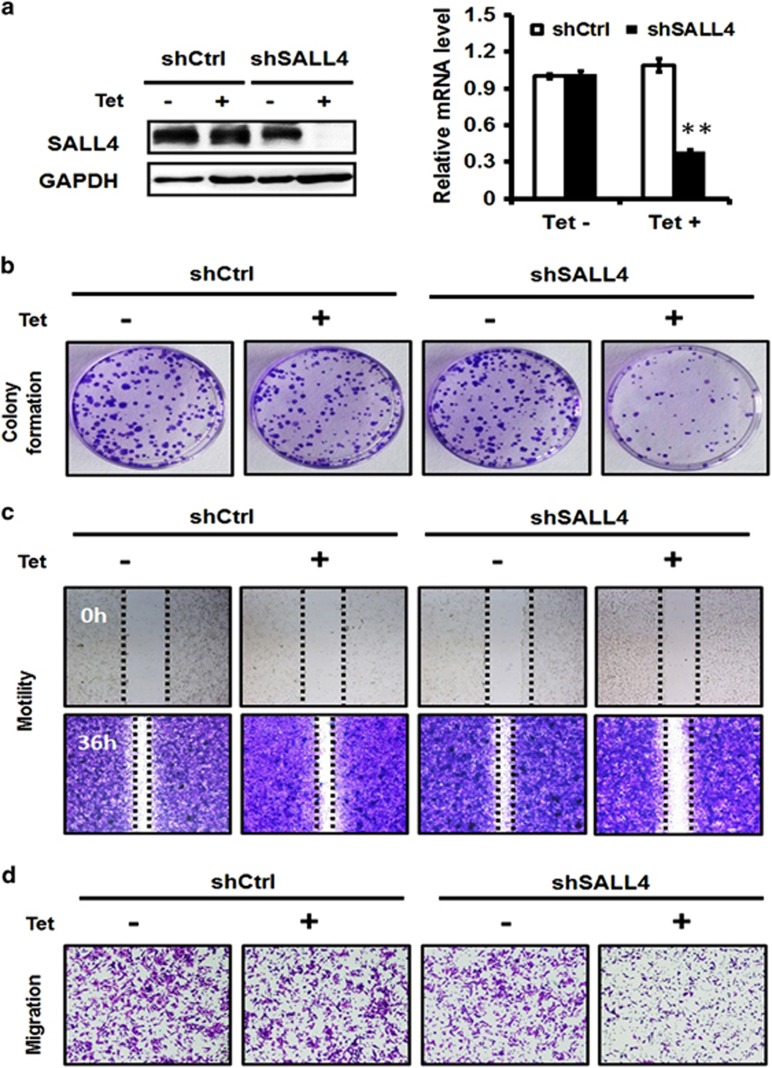
Inducible knockdown of SALL4 inhibits the proliferation and migration of gastric cancer cells. (**a**) Quantitative RT-PCR and western blot analyses of SALL4 expression in uninduced and tetracycline (Tet)-induced control and SALL4 knockdown gastric cancer cells. (**b**) Cell colony formation assay for uninduced and Tet-induced control and SALL4 knockdown gastric cancer cells. (**c**) Wound healing assay for uninduced and Tet-induced control and SALL4 knockdown gastric cancer cells. (**d**) Transwell migration assay for uninduced and Tet-induced control and SALL4 knockdown gastric cancer cells. **P<0.01, compared to uninduced group.

To further confirm the biological roles of SALL4 on gastric cancer cell proliferation, migration and invasion, we established an inducible SALL4-targeting shRNA-expressing gastric cancer cell line by using the Tet-on system. In the presence of tetracycline (Tet), SALL4 gene and protein expression decreased in gastric cancer cells as shown by the results of quantitative RT-PCR and western blot (Figure 2a). We next determined the effects of inducible SALL4 knockdown on gastric cancer cell proliferation, motility and migration. Compared to the uninduced cells, Tet-induced SALL4 knockdown cells generated less cell colonies (Figure 2b). Similarly, Tet-induced knockdown of SALL4 suppressed the motility and migration abilities of gastric cancer cells (Figures 2c and d). On the contrary, no significant changes were observed in the control cells with or without tetracycline treatment. Taken together, these results suggest that inducible knockdown of SALL4 also inhibits the proliferation and migration of gastric cancer cells.

### SALL4 knockdown inhibits the expression of stemness and EMT-related genes and suppresses the activation of ERK, STAT3 and NF-κB pathways

**Figure 3:**
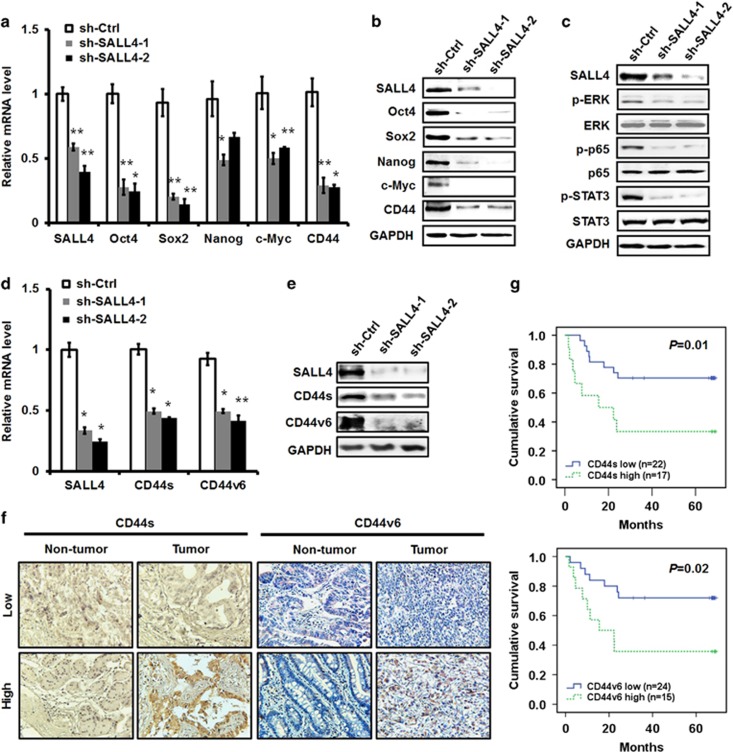
SALL4 knockdown reduces the expression of stemness and EMT-related genes and the activation of ERK, STAT3 and NF-κB pathways. (**a**) Quantitative RT-PCR analyses of Oct4, Sox2, Nanog, c-Myc and CD44 expression in control and SALL4 knockdown gastric cancer cells. (**b**) Western blot analyses of Oct4, Sox2, Nanog, c-Myc and CD44 expression in control and SALL4 knockdown gastric cancer cells. (**c**) Western blot analyses of p-ERK, ERK, p-p65, p65, p-STAT3 and STAT3 expression in control and SALL4 knockdown gastric cancer cells. (**d**) Quantitative RT-PCR analyses of CD44s and CD44v6 expression in control and SALL4 knockdown gastric cancer cells. (**e**) Western blot analyses of CD44s and CD44v6 expression in control and SALL4 knockdown gastric cancer cells. (**f**) CD44s and CD44v6 expression in tumour tissues and non-tumour tissues was detected in a tissue array by using immunohistochemistry. (**g**) The patients were divided into high or low group based on CD44s and CD44v6 expression. The overall survival probability was assessed by using the Kaplan–Meier curve. *P<0.05, **P<0.01, compared to sh-Ctrl group.

To elucidate the mechanism responsible for the inhibitory roles of SALL4 knockdown in gastric cancer cell proliferation, migration and invasion, we detected the expression of stemness and EMT-related genes since SALL4 has been previously suggested as a stemness and EMT regulator.^25^ The results of quantitative RT-PCR and western blot showed that SALL4 knockdown downregulated the expression of Oct4, Sox2, Nanog and c-Myc (Figures 3a and b), which have been previously shown to be the downstream targets of SALL4. In particular, we found that SALL4 knockdown inhibited the expression of CD44, a cell adhesion molecule, which has also been previously shown to be closely associated with cell stemness and EMT. Consistent with the results from stable knockdown experiments, inducible SALL4 knockdown also downregulated the expression of Oct4, Sox2, Nanog, c-Myc and CD44 (Supplementary Figure 3). We also found that stable and inducible knockdown of SALL4 upregulated the expression of E-cadherin (Supplementary Figure 4), which is in support of the finding that SALL4 promotes EMT in gastric cancer cells. We then detected the influence of SALL4 knockdown in the pathways that critically regulate tumour growth and metastasis. As shown in Figure 3c, SALL4 knockdown greatly inhibited the expression of phosphorylated ERK, STAT3 and NF-κB, suggesting an important role of SALL4 in modulating the activation of these pathways in gastric cancer cells.

To demonstrate whether SALL4 knockdown affects the expression of both standard and variant isoforms of CD44, we examined the expression of CD44s and CD44v6, a CD44 variant that is closely related to gastric cancer, in SALL4 knockdown gastric cancer cells. The results of quantitative RT-PCR and western blot showed that SALL4 knockdown resulted in the decreased expression of both CD44s and CD44v6 (Figures 3d and e), suggesting a common role of SALL4 in regulating CD44 family proteins. To reveal the clinical significance of CD44s and CD44v6 in gastric cancer, we examined CD44s and CD44v6 expression by using immunohistochemistry in a tissue array. The 39 gastric cancer patients were divided into high or low group according to the immunochemical staining results (Figure 3f). As shown in Figure 3g, high levels of CD44s and CD44v6 expression were associated with poor survival among the gastric cancer patients, indicating that CD44s and CD44v6 may serve as potential prognostic markers for gastric cancer.

### SALL4 upregulates CD44 expression in gastric cancer cells

**Figure 4:**
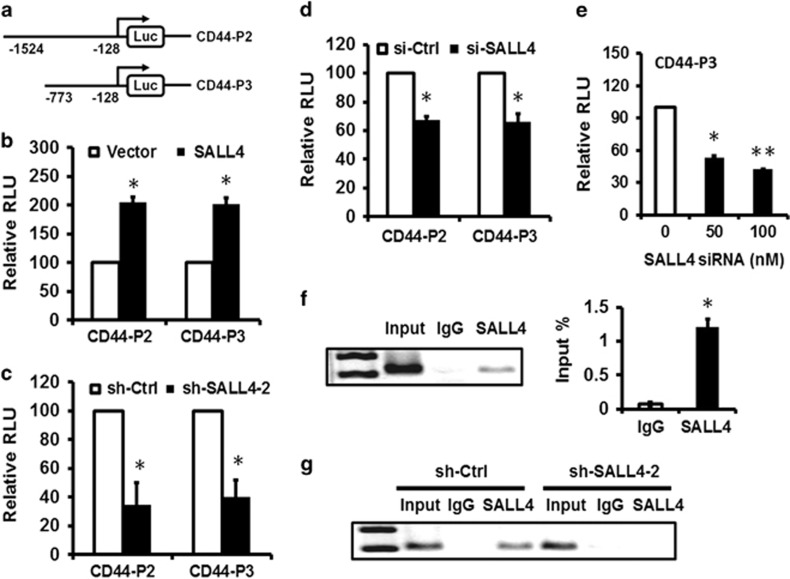
SALL4 regulates CD44 expression in gastric cancer cells. (**a**) Schematic image of luciferase reporter vectors under the control of CD44 promoter. (**b**) HEK293T cells were co-transfected with CD44 luciferase reporter and SALL4 plasmid. The luciferase activity was evaluated by using dual-luciferase reporter system. (**c**) SALL4 knockdown gastric cancer cells were transfected with CD44 luciferase reporter as indicated. The luciferase activity was evaluated by using dual-luciferase reporter system. (**d**) MGC80-3 cells were co-transfected with CD44 luciferase reporter and SALL4 siRNA. The luciferase activity was evaluated by using dual-luciferase reporter system. (**e**) MGC80-3 cells were co-transfected with CD44 luciferase reporter and different concentrations of SALL4 siRNA. The luciferase activity was evaluated by using dual-luciferase reporter system. (**f**) ChIP-PCR analyses of SALL4 binding to CD44 promoter in MGC80-3 cells. (**g**) ChIP-PCR analyses of SALL4 binding to CD44 promoter in control and SALL4 knockdown MGC80-3 cells. *P<0.05, **P<0.01.

Since CD44 showed a decreased expression in SALL4 knockdown gastric cancer cells, we then focused on the regulatory role of SALL4 in CD44 expression. To this end, we cloned CD44 promoter region and inserted it into a luciferase reporter vector (Figure 4a). The results of luciferase reporter assay showed that SALL4 overexpression upregulated while SALL4 knockdown downregulated the luciferase activity of CD44 promoter (Figures 4b and c). The −773 to −128 bp region in CD44 promoter was critical for SALL4-mediated transactivation. We further confirmed that SALL4 knockdown by siRNA downregulated the luciferase activity of CD44 promoter in a dose-dependent manner, suggesting an important regulatory role of SALL4 in CD44 gene expression (Figures 4d and e). We then performed a chromatin immunoprecipitation assay to test the binding of SALL4 protein to CD44 promoter in gastric cancer cells. The chromatin was immunoprecipitated by IgG or SALL4-specific antibodies for PCR detection. The results of ChIP assay showed an obvious enrichment of the chromatin in SALL4 group but not in IgG group (Figure 4f). To further confirm this, we compared the binding of SALL4 to the promoter region of CD44 gene between SALL4 knockdown cells and control cells. As shown in Figure 4g, SALL4 knockdown led to a decreased binding of SALL4 to the promoter region of CD44 gene. We also found a positive correlation between SALL4 and CD44 expression in gastric cancer cell lines and gastric cancer tissues (Supplementary Figure 5), further supporting the regulation of CD44 by SALL4. Taken together, these results suggest that SALL4 regulates the expression of CD44 in gastric cancer cells through binding to its promoter.

### CD44 rescue antagonizes SALL4 knockdown-mediated inhibition of the proliferation, migration and invasion of gastric cancer cells *in vitro*

**Figure 5:**
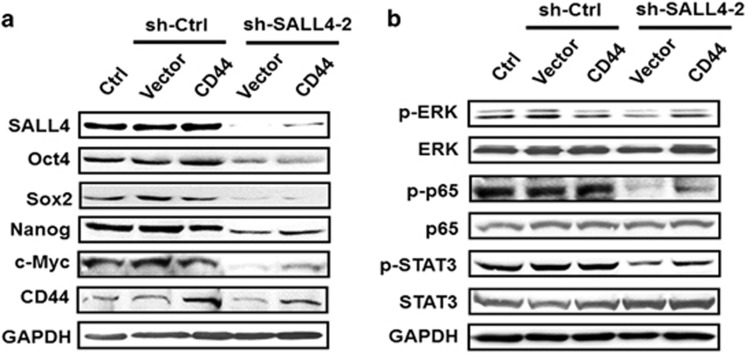
CD44 rescue restores the expression of stemness and EMT-related genes and re-activates ERK, STAT3 and NF-κB pathways in SALL4 knockdown gastric cancer cells. (**a**) Western blot analyses of Oct4, Sox2, Nanog, c-Myc and CD44 expression in control and SALL4 knockdown gastric cancer cells with or without CD44 overexpression. (**b**) Western blot analyses of p-ERK, ERK, p-p65, p65, p-STAT3 and STAT3 expression in control and SALL4 knockdown gastric cancer cells with or without CD44 overexpression.

**Figure 6:**
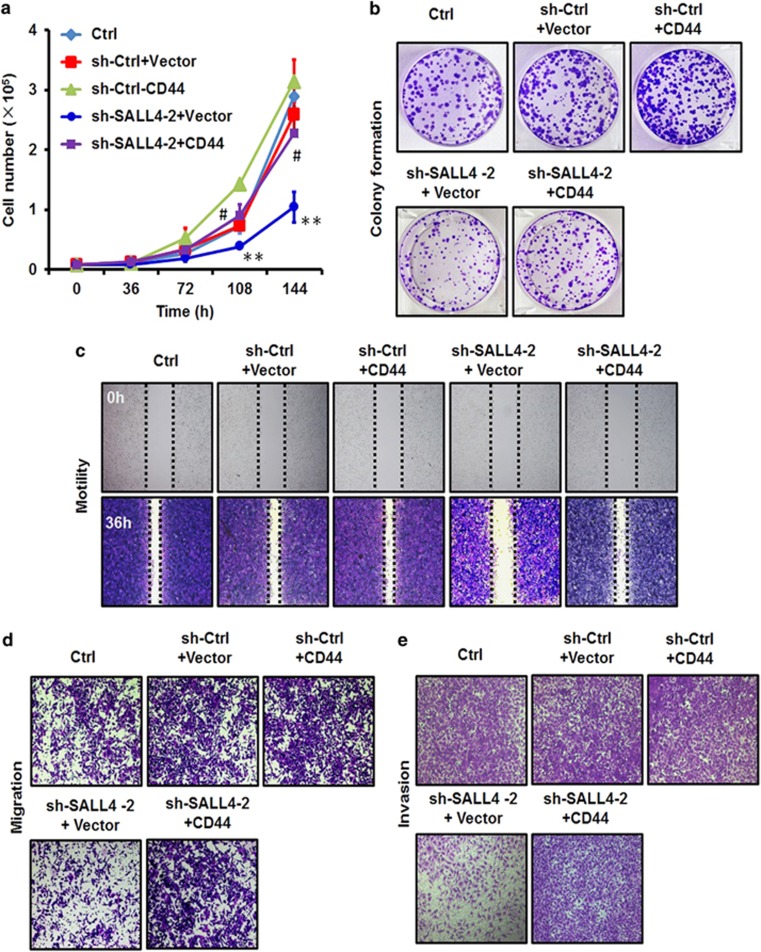
CD44 rescue antagonizes SALL4 knockdown-mediated inhibition of the proliferation, migration and invasion of gastric cancer cells *in vitro*. (**a**) Cell counting assay for control and SALL4 knockdown gastric cancer cells with or without CD44 overexpression. (**b**) Cell colony formation assay for control and SALL4 knockdown gastric cancer cells with or without CD44 overexpression. (**c**) Wound healing assay for control and SALL4 knockdown gastric cancer cells with or without CD44 overexpression. (**d**) Transwell migration assay for control and SALL4 knockdown gastric cancer cells with or without CD44 overexpression. (**e**) Matrigel invasion assay for control and SALL4 knockdown gastric cancer cells with or without CD44 overexpression. **P<0.01, compared to sh-Ctrl+Vector group; ^#^*P*<0.05, compared to sh-SALL4-2+Vector group.

We next performed the rescue study to determine the importance of CD44 regulation to the oncogenic roles of SALL4 in gastric cancer. The efficiency of SALL4 knockdown was determined by using quantitative RT-PCR and western blot (Figure 5a; Supplementary Figure 6). The restoration of CD44 expression in SALL4 knockdown cells was verified by using western blot (Figure 5a). We found that CD44 overexpression re-activated ERK, STAT3 and NF-κB pathways in SALL4 knockdown cells (Figure 5b). We next determined the roles of CD44 rescue in the proliferation, migration and invasion of SALL4 knockdown cells. The results of cell counting assay showed that SALL4 knockdown decreased the growth of gastric cancer cells while CD44 overexpression significantly increased the growth of gastric cancer cells (Figure 6a). The results of cell colony formation assay further confirmed that CD44 overexpression increased the colony formation ability of SALL4 knockdown cells (Figure 6b). In addition, the results of wound healing assay showed that CD44 overexpression rescued the decreased motility of SALL4 knockdown cells (Figure 6c). Furthermore, the results of transwell migration and matrigel invasion assays showed that CD44 overexpression rescued the reduced migration and invasion abilities of SALL4 knockdown cells (Figures 6d and e). In support of these observations, we found that SALL4 knockdown upregulated the expression of E-cadherin while decreased the expression of N-cadherin; however, CD44 overexpression led to the opposite effects on SALL4 knockdown cells (Supplementary Figure 7). Taken together, these results indicate that CD44 overexpression antagonizes SALL4 knockdown-mediated inhibition of the proliferation, migration and invasion of gastric cancer cells *in vitro*.

### CD44 rescue antagonizes SALL4 knockdown-mediated inhibition of tumour growth *in vivo*

**Figure 7:**
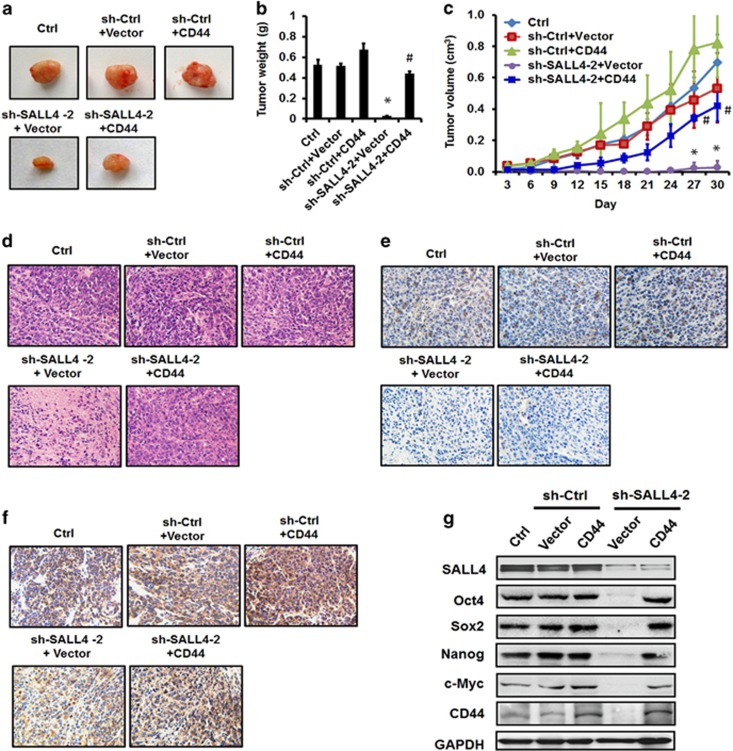
CD44 rescue antagonizes SALL4 knockdown-mediated inhibition of tumour growth and metastasis *in vivo*. (**a**) Representative images of tumours from mice injected with control and SALL4 knockdown gastric cancer cells with or without CD44 overexpression. (**b**) The mean weight of tumours from mice injected with control and SALL4 knockdown gastric cancer cells with or without CD44 overexpression. (**c**) The mean volume of tumours from mice injected with control and SALL4 knockdown gastric cancer cells with or without CD44 overexpression. (**d**) HE staining of tumour tissue sections from mice injected with control and SALL4 knockdown gastric cancer cells with or without CD44 overexpression. (**e**) Immunohistochemical staining for SALL4 in tumour tissue sections from mice injected with control and SALL4 knockdown gastric cancer cells with or without CD44 overexpression. (**f**) Immunohistochemical staining for CD44 in tumour tissue sections from mice injected with control and SALL4 knockdown gastric cancer cells with or without CD44 overexpression. (**g**) Western blot analyses of Oct4, Sox2, Nanog, c-Myc and CD44 expression in control and SALL4 knockdown gastric cancer cells with or without CD44 overexpression. *P<0.05, compared to sh-Ctrl+Vector group; ^#^*P*<0.05, compared to sh-SALL4-2+Vector group.

To further clarify the importance of SALL4-mediated upregulation of CD44 in gastric cancer growth *in vivo*, we implanted SALL4 knockdown cells with or without CD44 overexpression into the nude mice. The results of tumour growth curve and tumour weight showed that SALL4 knockdown significantly retarded the growth of xenograft tumours in mice; however, the simultaneous overexpression of CD44 greatly accelerated xenograft tumour growth (Figures 7a–c). The results of hematoxylin-eosin staining further confirmed the increased malignance in SALL4 knockdown cells with CD44 overexpression (Figure 7d). The expression of SALL4 and CD44 proteins in different groups was confirmed by using immunohistochemical staining (Figures 7e). Consistent with that observed *in vitro*, CD44 overexpression reversed the down-regulation of Oct4, Sox2, Nanog and c-Myc by SALL4 knockdown *in vivo* (Figure 7g). We also confirmed the re-activation of ERK, STAT3 and NF-κB pathways by CD44 overexpression in SALL4 knockdown cells *in vivo* (Supplementary Figure 8). Taken together, these results indicate that CD44 overexpression antagonizes SALL4 knockdown-mediated inhibition of tumour growth *in vivo*.

## Discussion

In this study we demonstrated that SALL4, a stem cell factor, promoted gastric cancer progression by activating CD44 expression. We provided evidence that stable and inducible knockdown of SALL4 greatly reduced the proliferation, migration and invasion of gastric cancer cells and downregulated the expression of EMT and stemness-related genes. In particular, we identified CD44 as a downstream target of SALL4 in gastric cancer cells. SALL4 bound to the promoter region of CD44 gene and transcriptionally activated its expression. We further confirmed that CD44 overexpression could antagonize SALL4 knockdown-mediated inhibition of gastric cancer cell proliferation, migration and invasion *in vitro* and gastric cancer growth *in vivo*, suggesting that CD44 contributed, at least in part, to SALL4-mediated gastric cancer growth and metastasis. Although the association of SALL4 and CD44 expression in cancer has been previously described,^14^ to our best knowledge, this is the first report to suggest CD44 as a direct target of SALL4 and to reveal the functional importance of CD44 regulation to the oncogenic roles of SALL4 in gastric cancer.

SALL4 is a newly identified oncogene that is involved in tumorigenesis, tumour growth, tumour metastasis and drug resistance through the regulation of various downstream genes.^30^ In AML, SALL4 directly interacts with β-catenin and induces the activation of Wnt/β-catenin signalling pathway, leading to the upregulation of the target genes of Wnt/β-catenin pathway including cyclin D1 and c-Myc.^7^ CD44 is also suggested as a downstream target gene of Wnt/β-catenin signalling. Thus, it could not be excluded that SALL4 might also activate CD44 expression through the interaction with β-catenin. In addition, SALL4 could activate its downstream targets by recruiting a histone methyltransferase complex to specific promoter regions to mediate H3-K4 trimethylation and transcription activation.^11^ Yang *et al* suggest that SALL4 binds to the promoter region of Bmi-1 and triggers high levels of histone methylation in hematopoietic and leukemic cells.^10^ Li *et al.* suggest that SALL4 interacts with mixed-lineage leukemia, a histone methyltransferase, and co-occupies the HOXA9 promoter region with mixed-lineage leukemia in AML cells.^9^ Therefore, the methylated status of CD44 gene promoter in gastric cancer cells that harbour high level of SALL4 deserves further investigation.

CD44 is a cell surface adhesion molecule expressed on a variety of cells and is involved in cell proliferation, differentiation, adhesion, migration and invasion. CD44 is critical for EMT and cancer development.^31, 32^ CD44 has also been identified as a cancer stem cell marker.^33^ CD44 expression is elevated in gastric cancer and is positively correlated with tumour stage and tumour metastasis, serving as an independent prognostic factor for gastric cancer.^34, 35^ While both SALL4 and CD44 play important roles in gastric cancer, the connection between them has not been investigated. In this study, we observed the downregulation of CD44 in stable and inducible SALL4 knockdown gastric cancer cells. Moreover, overexpression of CD44 in SALL4 knockdown cells led to increased gastric cancer cell proliferation, migration and invasion *in vitro* as well as increased tumour growth in mouse models. Clinical studies demonstrated that the expression of SALL4 and CD44 was positively correlated in gastric cancer patient samples. Consistent with our previous findings showing that SALL4 overexpression is associated with poor prognosis, the elevated expression of CD44 also showed a worse overall survival in gastric cancer patients, indicating an important role of SALL4-CD44 signalling pathway in gastric cancer development and progression. ChIP studies showed that the endogenous SALL4 protein could bind to the specific promoter region of CD44 in gastric cancer cells, suggesting that CD44 is a direct target of SALL4. The specific binding site at the promoter regions of SALL4 target genes has not been well characterized. We have compared the potential SALL4 binding site at the promoter regions of ABCA3,^12^ HOXA9^9^ and Oct4^1^ with that of CD44 and observed a consensus ‘GAAG' nucleotide sequence at the promoter regions of these genes. Thus, further study using site mutagenesis will help confirm the exact binding site for SALL4 at the promoter regions of its target genes.

In conclusion, our findings suggest that CD44 is a downstream target gene of SALL4 and is partially responsible for the oncogenic roles of SALL4 in gastric cancer. Our findings provide a novel insight into the mechanism responsible for the oncogenic function of SALL4 in cancer, suggesting that targeted depletion of the SALL4-CD44 pathway may be a novel avenue for anti-cancer therapy.

## Materials and methods

### Cell culture

Human gastric cancer cell line MGC80-3 and human embryonic kidney cell line 293 T were purchased from the Institute of Biochemistry and Cell Biology at the Chinese Academy of Sciences (Shanghai, China). Cells were cultured in high-glucose DMEM (Dulbecco's modified Eagle's medium; Gibco, Grand Island, NY, USA) supplemented with 10% fetal bovine serum (FBS; Gibco) at 37 °C in humidified air with 5% CO_2_. Cells have been regularly tested for Mycoplasma and were free of this contamination.

### Gene silencing and gene overexpression

The SALL4-targeting shRNA lentivirus was provided by Genechem (Shanghai, China). GFP served as a reporter gene in the lentiviral vector. Cells were transfected with lentivirus at an MOI (multiplicity of infection) of 100 for 24 h and then selected with puromycin (0.8 μg/ml) for 3 days. The scramble and SALL4 siRNAs were synthesized by Genepharma (Shanghai, China). Cells were seeded in six-well plates at a density of 1 × 10^5^ cells/well and transiently transfected with siRNAs by using Lipofectin reagent (Invitrogen, Carlsbad, CA, USA). The sequences of SALL4 shRNA and siRNA were listed in Supplementary Table 1. Human CD44 gene (NM_001001389.1) overexpressing lentivirus was provided by Cyagen Biosciences (Guangzhou, China). Cells were transfected with the CD44-overexpressing lentivirus at an MOI of 200 for 24 h.

### Inducible gene knockdown

The inducible SALL4 knockdown lentiviral vector was generated by ligating the Tet-pLKO-puro vector with SALL4 shRNA oligos. The recombinant lentivirus was produced by co-transfecting HEK293T cells with pLKO-GFP-shRNA or pLKO-SALL4-shRNA, PU1562, and PU1563 plasmids by using Lipofectin reagent. The virus-containing supernatant was harvested at 48 and 72 h post-transfection. MGC80-3 cells were transduced with the prepared lentivirus and stable cell lines were obtained after selection with 0.8 μg/ml of puromycin for 3 days. The expression of shRNA was induced by the addition of 5 μg/ml tetracycline (Tet). SALL4 knockdown was validated by using quantitative RT-PCR and western blot. SALL4 shRNA oligos were synthesized by Invitrogen and the sequences were listed in Supplementary Table 1.

### RNA extraction, RT-PCR and quantitative RT-PCR

Total RNA were extracted from the cells by using Trizol reagent (Invitrogen) and one microgram of RNA was reverse transcribed to cDNA by using reverse transcriptase (Vazyme, Nanjing, China). Quantitative PCR was performed by using a SYBR Green I real-time detection kit (Cwbio, Beijing, China) on a Bio-Rad CFX96 detection system. The relative gene expression was normalized to β-actin. The primers specific for target genes were listed in Supplementary Table 2.

### Western blot

The cells were lysed in RIPA buffer containing proteinase inhibitors. Equal amount of proteins was loaded and separated on a 12% SDS-PAGE gel. Following electrophoresis, the proteins were transferred to a PVDF (polyvinylidene difluoride) membrane, blocked in 5% (w/v) non-fat milk, and incubated with the primary antibodies at 4 ^o^C overnight. The sources of primary antibodies were: SALL4 (954-1053; Abnova, Taipei, Taiwan); CD44 (BS6825; Bioworld technology, Louis Park, MN, USA) and GAPDH (MB001, Bioworld technology); ERK (4695S; Cell Signaling Technology, Beverly, MA, USA), p-ERK (4370S; Cell Signaling Technology), NF-κB p65 (8242S; Cell Signaling Technology), p-NF-κB p65 (3033P; Cell Signaling Technology), STAT3 (4904P; Cell Signaling Technology), p-STAT3 (9145P; Cell Signaling Technology), N-cadherin (13116S; Cell Signaling Technology), and Oct4 (2750S; Cell Signaling Technology); Sox2 (AB5603; Merck Millipore, Shanghai, China), c-Myc (10057-1-AP; Proteintech, Rosemont, IL, USA), Nanog (AF1505; Signalway Antibody, College Park, MD, USA), E-cadherin (H-108, Santa Cruz Biotechnology, Dallas, TX, USA). After washing with TBS/T for three times, the membranes were incubated with HRP-conjugated goat anti-rabbit or anti-mouse secondary antibodies (Bioworld technology) at room temperature for 1 h. The protein bands were visualized by enhanced chemiluminescence.

### Cell counting and colony formation assays

The stably transfected cells were seeded into 24-well plate (8000 cells/well) and cultured under standard conditions. Cells were collected and counted at the indicated time points. The stably transfected cells were seeded into six-well plates at a density of 500 cells per well. After continuous incubation for 10 days, the cells were fixed with 4% paraformaldehyde and stained with 1% crystal violet for 15 min. All the experiments were performed in triplicates.

### Cell apoptosis assay and cell cycle analysis

The stably transfected cells were harvested, washed with phosphate-buffered saline, and subjected to Annexin V/PI double staining (Invitrogen). The percentage of apoptotic cells was detected by flow cytometric analysis. The stably transfected cells were collected and washed with phosphate-buffered saline twice, and then stained with propidium iodide for 30 min (Invitrogen). The cell cycle distribution of the stained cells was assessed on a flow cytometer (BD FACS Calibur).

### Wound healing assay

The confluent cell monolayers were wounded by scratching with a 10 μl pipette tip and then cultured for 36 h. Cell migration over the scraped area was photographed at 0 and 36 h. At the end of the experiment, the cells were fixed with 4% paraformaldehyde and stained with 1% crystal violet for 15 min.

### Transwell migration assay

Cells were plated into the top chamber at a density of 1 × 10^5^/well in serum-free medium. The complete medium was placed into the bottom chamber. After incubation at 37 °C in 5% CO_2_ for 12 h, the cells remaining at the upper surface of the membrane were removed with a cotton swab. The cells that migrated through the 8-μm sized pores and adhered to the lower surface of the membranes were fixed with 4% paraformaldehyde, stained with crystal violet and photographed under a light microscope.

### Cell invasion assay

The matrigel (BD Biosciences, San Jose, CA, USA) was diluted with serum-free medium (1:3) and 50 microlitres of the diluted matrigel were added into the upper chamber followed by incubation at 37 °C for 1 h. Cells suspended in serum-free medium were seeded into the upper chamber containing coagulated matrigel. The complete medium was placed into the bottom chamber. Cells were incubated at 37 °C for 36 h to allow the cells to invade into the lower membrane through matrigel. At the end of the experiment, the invaded cells were fixed with 4% paraformaldehyde, stained with crystal violet and photographed under a light microscope.

### Luciferase reporter assay

CD44 promoter was cloned into the pGL3-Basic vector by proof-reading PCR. For the luciferase reporter assay, HEK293T cells or MGC80-3 cells were co-transfected with CD44 promoter luciferase reporter and SALL4 plasmid or SALL4 siRNA as indicated. The Renilla luciferase reporter was used as internal control. The activities of firefly luciferase and Renilla luciferase were quantified by using the dual luciferase reporter assay system (Promega, Madison, WI, USA).

### Chromatin immunoprecipitation assay

The chromatin immunoprecipitation assay was performed in MGC80-3 cells by using a commercial kit (Millipore, Darmstadt, Germany). After cross-linking with 1% formaldehyde at 37 °C for 10 min, the cells were harvested in sodium dodecyl sulfate lysis buffer and the DNA was shredded to fragments of 200 bp by sonication. The pre-cleared chromatin was incubated with the antibodies against SALL4 or non-specific IgG overnight. Protein G-agarose beads were added and incubated at 4 °C for 1 h. After reversing the cross-links, the DNA was isolated and used for PCR. The specific primers for PCR detection of the responsive element in CD44 promoter were shown in Supplementary Table 3.

### Animal model

Male BALB/c nude mice aged 4 weeks were purchased from the Laboratory Animal Center of Shanghai at the Academy of Science (Shanghai, China). The mice were randomly divided into five groups (five mice/group) as indicated. Cells (2.0 × 10^6^ per mice) suspended in 100 μl phosphate-buffered saline were implanted subcutaneously into the right flanks of the mice. The nude mice were regularly fed and the tumours were measured twice a week. The tumour volume was calculated by using the following formula: V(cm^3^)=1/2 × length × width^2^. The animal studies were approved by the Institutional Animal Care and Use Committee of Jiangsu University.

### Tissue array and immunohistochemistry

Tissue array was purchased from Shines Pharmaceuticals (Shanghai, China). A total of 39 pairs of tumour tissues and non-tumour tissues were included in the tissue array. Tissue array was incubated with antibodies against CD44s (MAB7045; R&D Systems, Minneapolis, MN, USA) and CD44v6 (AB2080; Millipore, Billerica, MS, USA). Immunohistochemical staining was performed as previously described.^36^ IHC scoring was assessed by two pathologists in a double-blinded manner. For animal studies, the mice were sacrificed at 5 weeks after tumour cell implantation and the tumour tissues were excised. The paraffin-embedded tissue sections were made and used for HE staining and immunohistochemical staining of SALL4 and CD44.

### Statistical analysis

All the data were shown as mean±standard deviation (s.d.). The statistically significant differences between groups were assessed by analysis of variance (ANOVA) or *t*-test using SPSS (Version 22.0, Chicago, IL, USA). The Kaplan–Meier curve was used to determine survival probability and differences were assessed by the log-rank test. *P* value <0.05 was considered significant.
