# Supplementary material for: SALL4 promotes gastric cancer progression through activating CD44 expression
Source: Oncogenesis. 2016 Nov 7;5(11):e268–. doi: 10.1038/oncsis.2016.69 (PMC5141291; doi:10.1038/oncsis.2016.69)
Supplement: Supplementary Information [file oncsis201669x1.docx]

**Supplementary Figure Legend**

**Supplementary Figure 1.** Quantitative RT-PCR analyses of SALL4A and SALL4B expression in control and SALL4 knockdown gastric cancer cells. **P*<0.05, ***P*<0.01, compared to sh-Ctrl group.

**Supplementary Figure 2.** MTT assay for the sensitivity of control and SALL4 knockdown gastric cancer cells to cisplatin treatment. ***P*<0.01, compared to sh-Ctrl group.

**Supplementary Figure 3.** Quantitative RT-PCR analyses of Oct4, Sox2, Nanog, c-Myc, and CD44 expression in uninduced and Tet-induced SALL4 knockdown gastric cancer cells. **P*<0.05, ***P*<0.01, compared to sh-Ctrl group.

**Supplementary Figure 4.** Quantitative RT-PCR analyses of E-cadherin expression in stable (**a**) and inducible (**b**) SALL4 knockdown gastric cancer cells. **P*<0.05.

**Supplementary Figure 5.** (**a**) Western blot analyses of SALL4 and CD44 expression in MGC80-3 and HGC-27 cells. (**b**) Quantitative RT-PCR analyses of SALL4 and CD44 expression in 10 paired gastric cancer tissues and adjacent noncancerous tissues.

**Supplementary Figure 6.** Quantitative RT-PCR analyses of SALL4 expression in SALL4 knockdown cells with or without CD44 overexpression. **P*<0.05, compared to sh-Ctrl+Vector group;

**Supplementary Figure 7.** Western blot analyses of E-cadherin and N-cadherin expression in SALL4 knockdown cells with or without CD44 overexpression.

**Supplementary Figure 8.** Western blot analyses of p-ERK, ERK, p-p65, p65, p-STAT3, and STAT3 expression in SALL4 knockdown cells with or without CD44 overexpression.
