# Supplementary figures and images for: SALL4 promotes gastric cancer progression through activating CD44 expression
Source: Oncogenesis. 2016 Nov 7;5(11):e268–. doi: 10.1038/oncsis.2016.69 (PMC5141291; doi:10.1038/oncsis.2016.69)

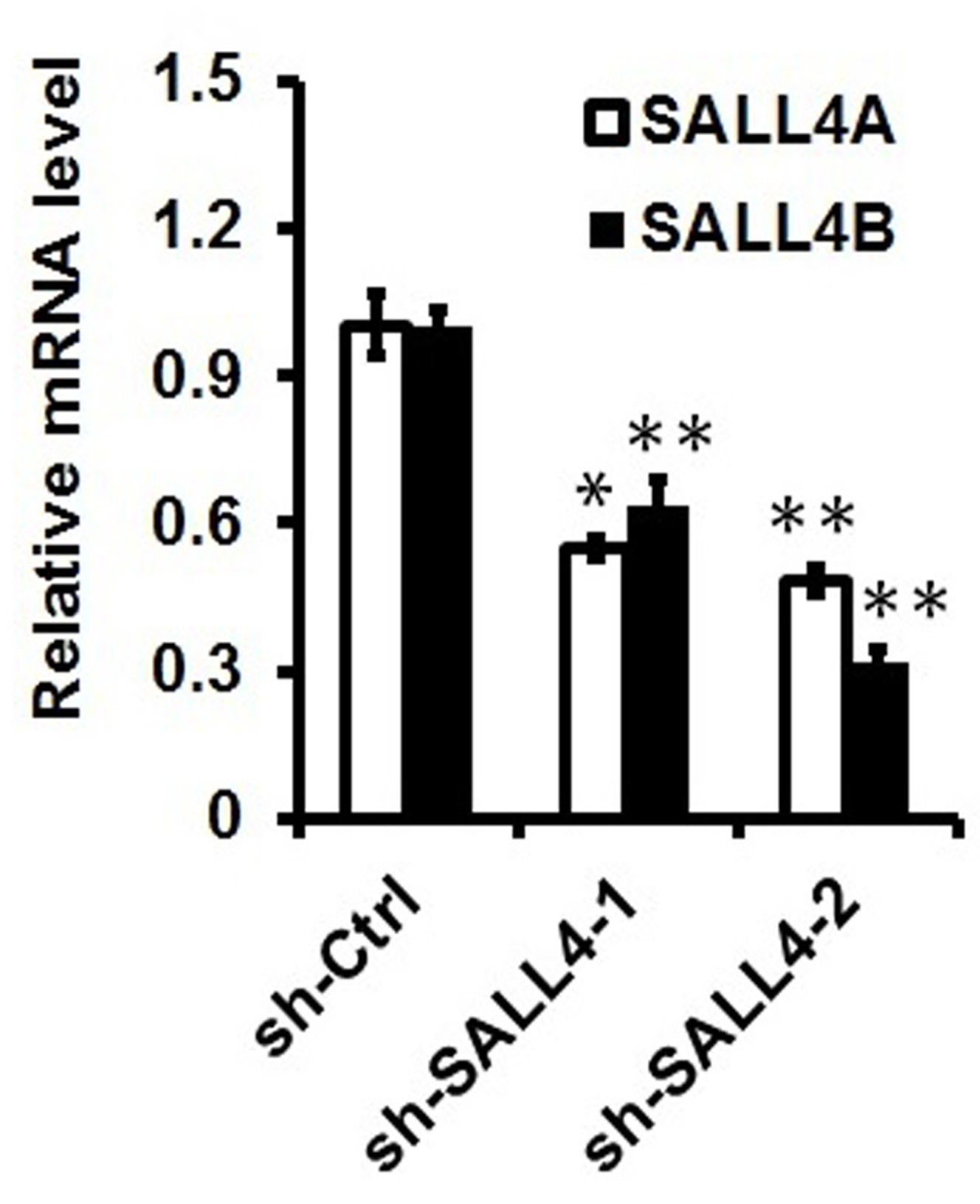

Supplement: Supplementary Figure 1 [file oncsis201669x2.tif]

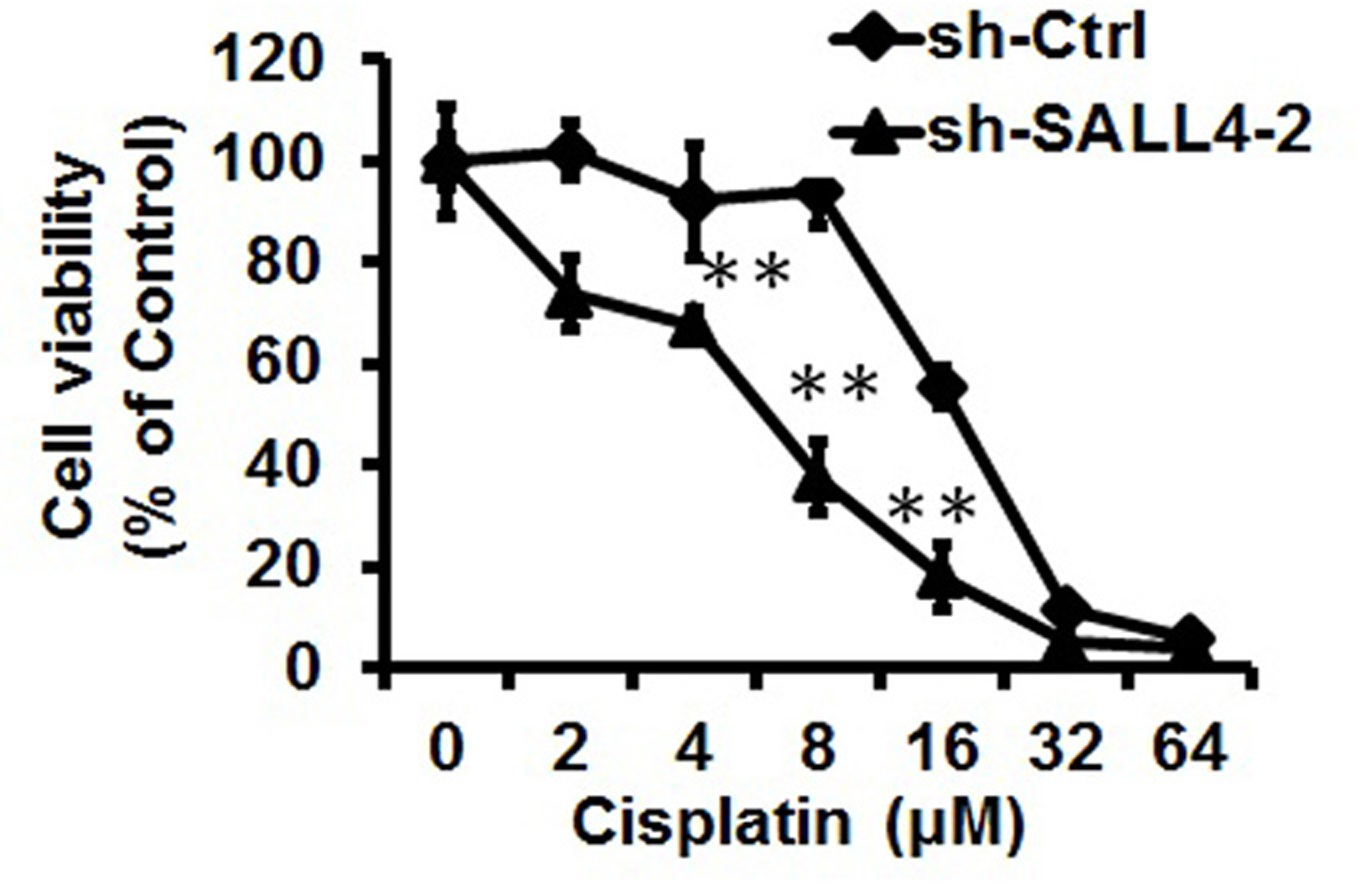

Supplement: Supplementary Figure 2 [file oncsis201669x3.tif]

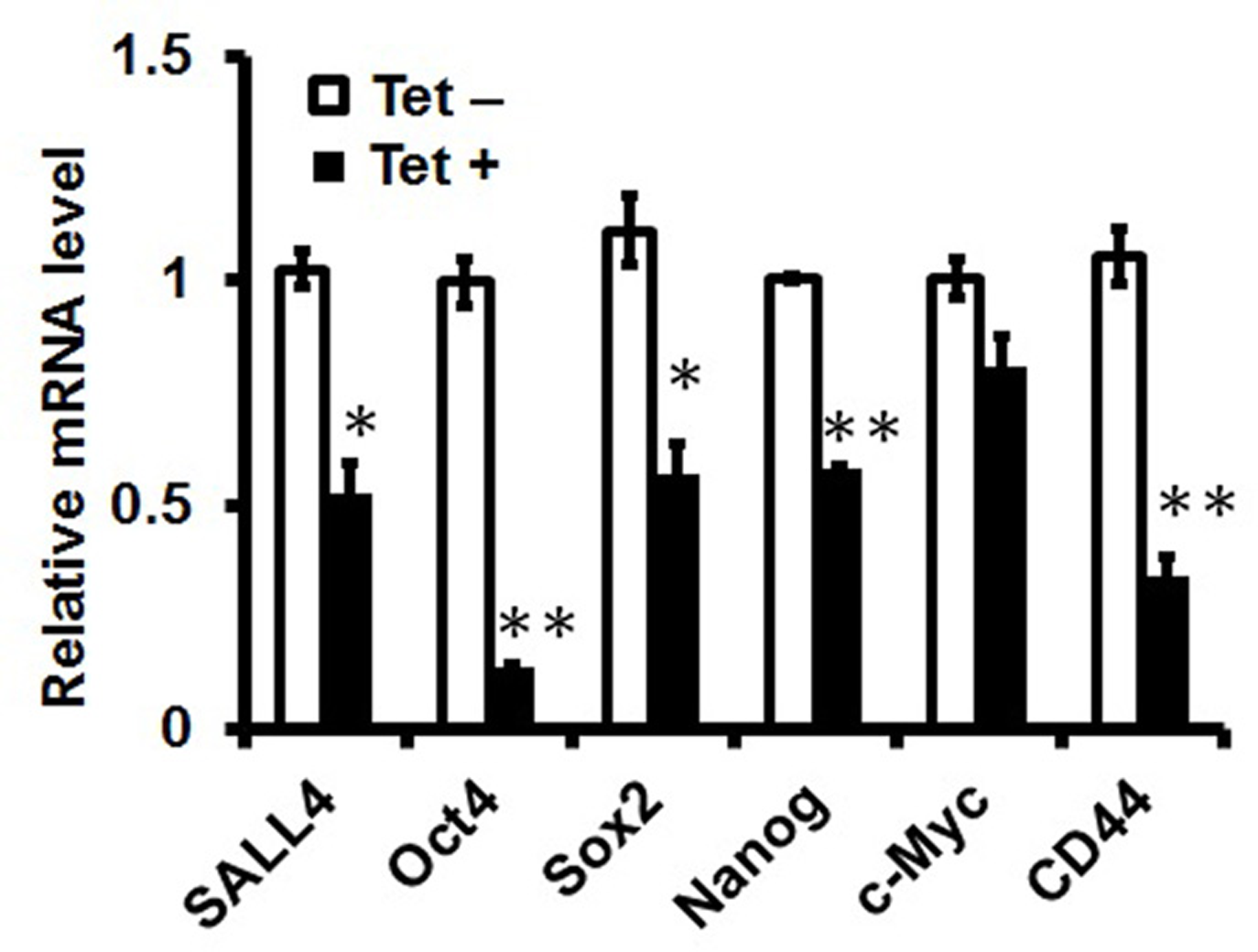

Supplement: Supplementary Figure 3 [file oncsis201669x4.tif]

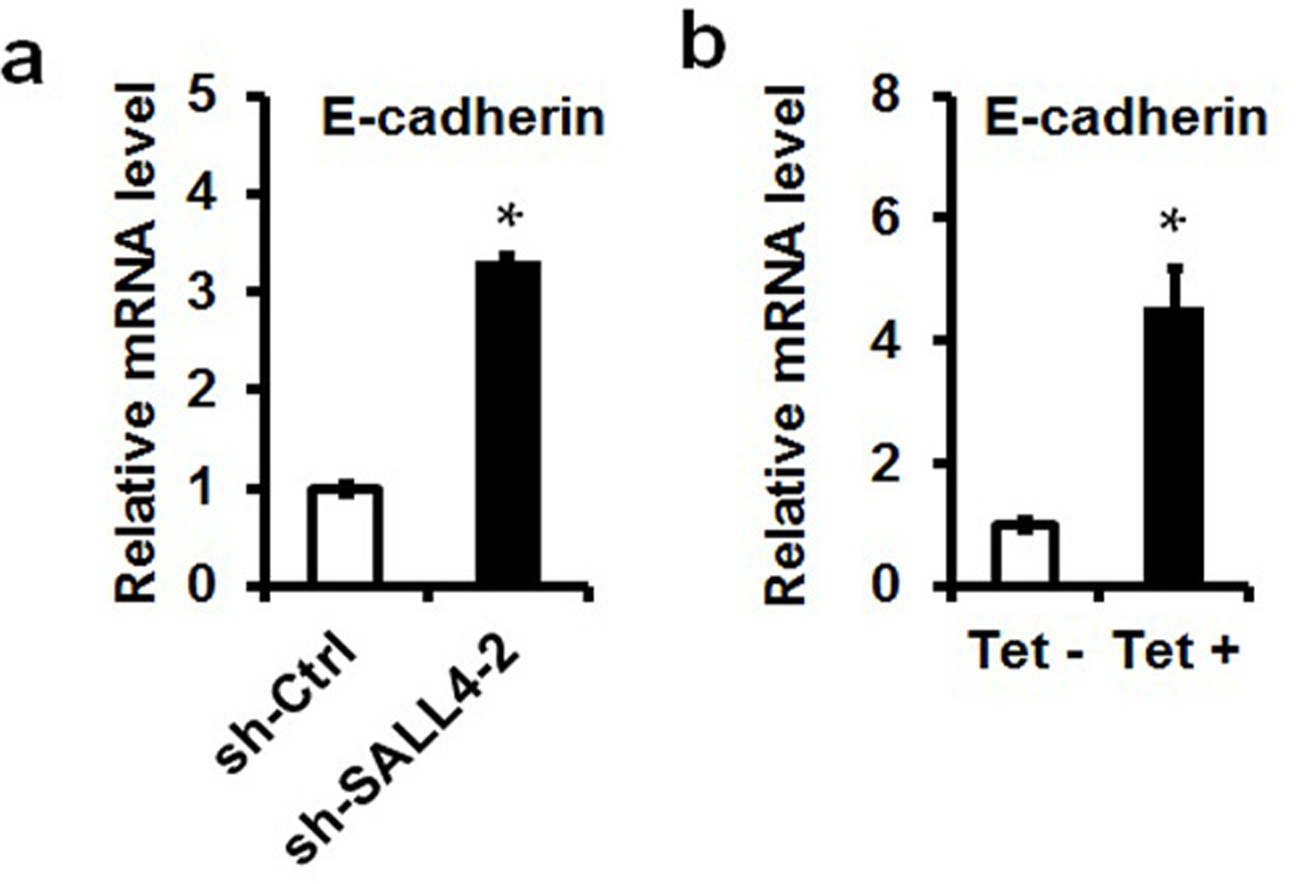

Supplement: Supplementary Figure 4 [file oncsis201669x5.tif]

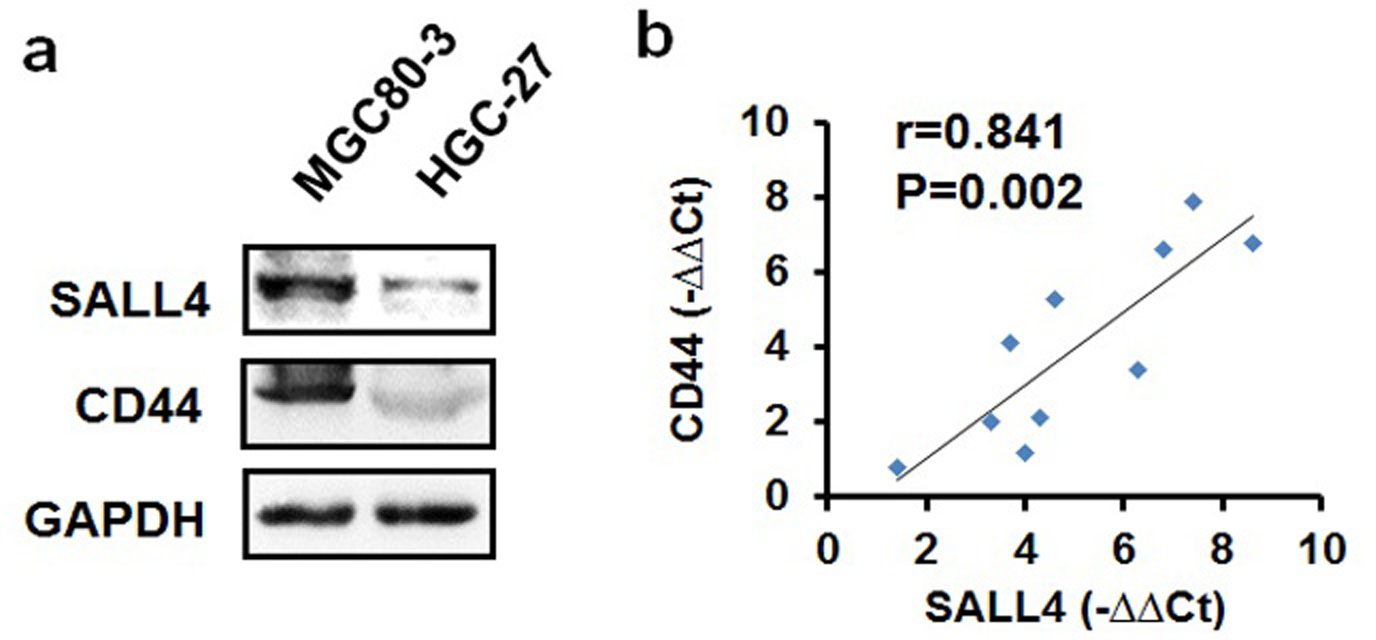

Supplement: Supplementary Figure 5 [file oncsis201669x6.tif]

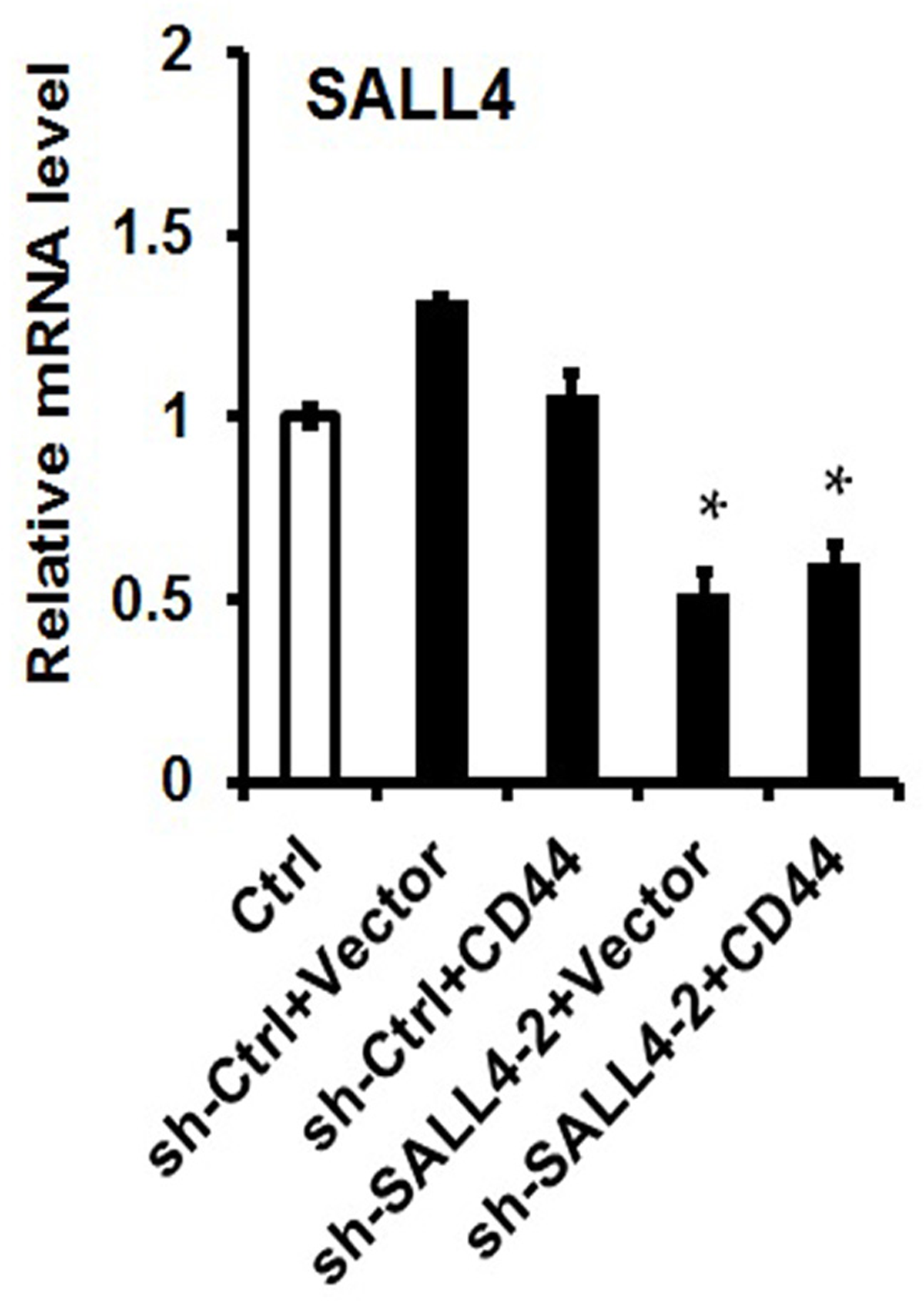

Supplement: Supplementary Figure 6 [file oncsis201669x7.tif]

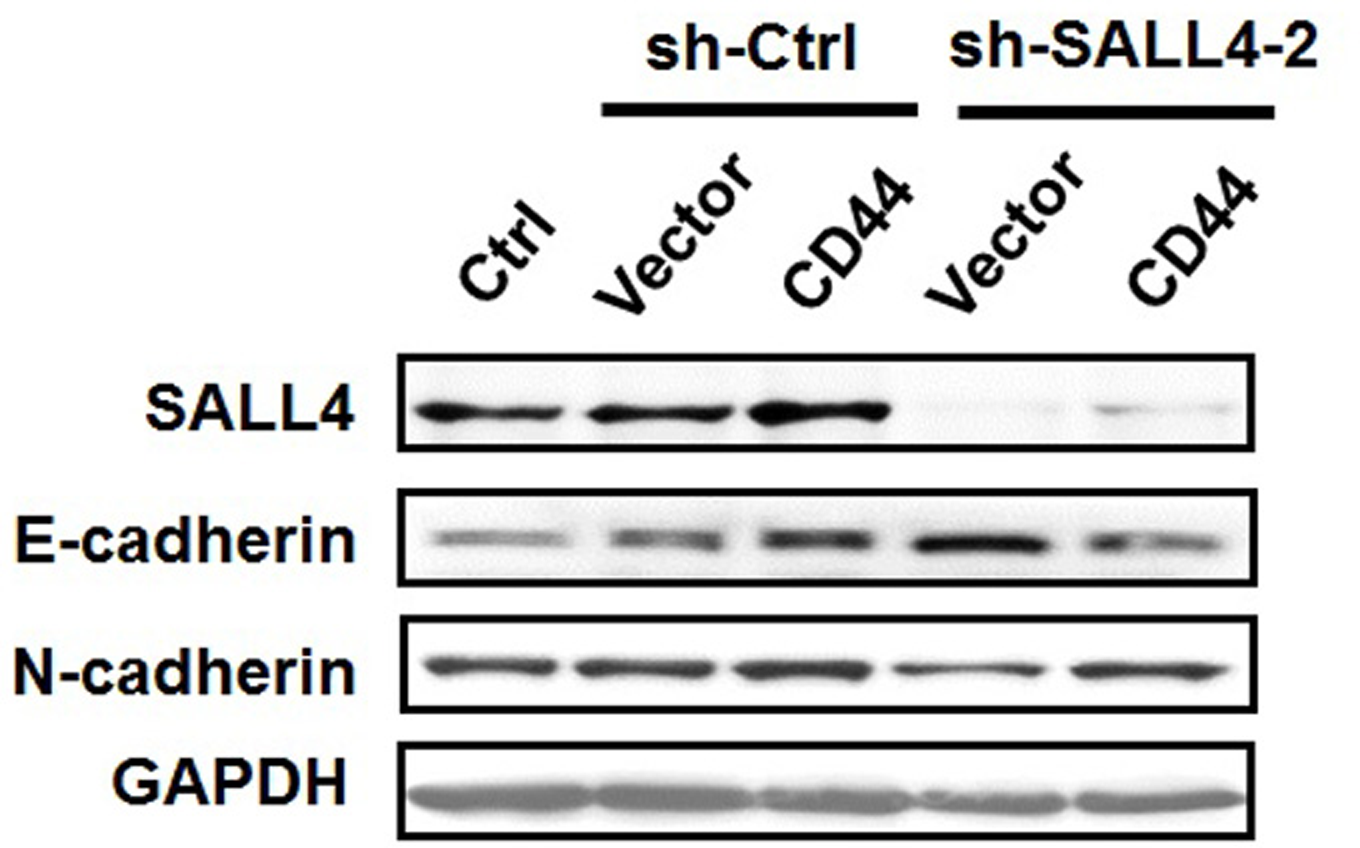

Supplement: Supplementary Figure 7 [file oncsis201669x8.tif]

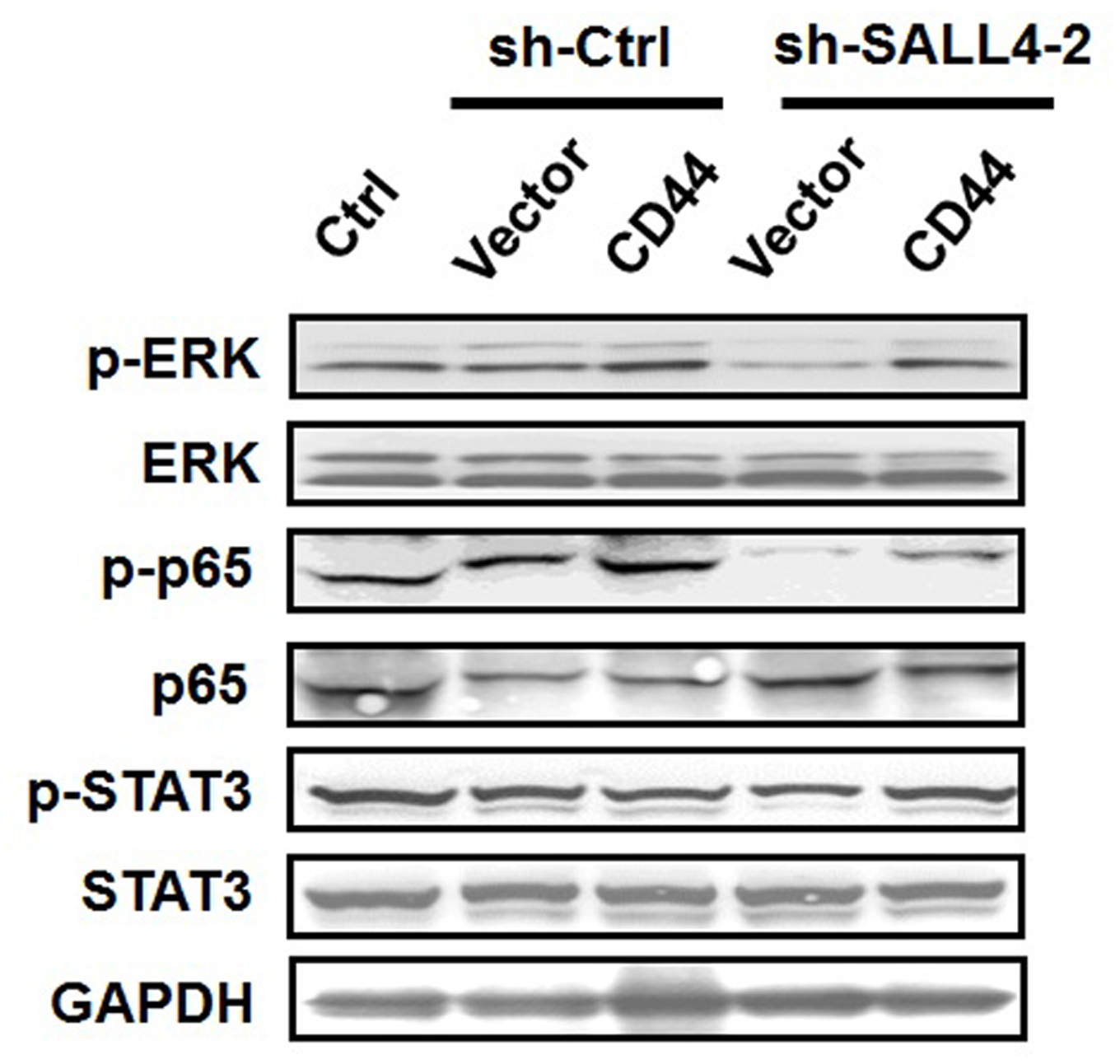

Supplement: Supplementary Figure 8 [file oncsis201669x9.tif]
