# Supplementary material for: SALL4 promotes gastric cancer progression through activating CD44 expression
Source: Oncogenesis. 2016 Nov 7;5(11):e268–. doi: 10.1038/oncsis.2016.69 (PMC5141291; doi:10.1038/oncsis.2016.69)
Supplement: Supplementary Table 1 [file oncsis201669x10.docx]

**Supplemental Table 1.** The sequences of SALL4 knockdown shRNA and siRNA

| shRNA | Target sequence |
| --- | --- |
| sh-Ctrl | 5’-TTCTCCGAACGTGTCACGT-3’ |
| sh-SALL4-1 | 5’-GACCTATGTCAAGGTTGAA-3’ |
| sh-SALL4-2 | 5’-GAGGATGAAGCCACAGTAA-3’ |
| si-Ctrl | F: 5’-UUCUCCGAACGUGUCACGUTT-3’  R: 5’-ACGUGACACGUUCGGAGAATT-3’ |
| si-SALL4 | F: 5’-GUCUCUGGAUGCCUUGAAATT-3’  R: 5’-UUUCAAGGCAUCCAGAGACTT-3’ |
| shRNA oligos | F: 5’-CCGGGCCTTGAAACAAGCCAAGCTACTCGAGTAGCTT  GGCTTGTTTCAAGGCTTTTTG-3’  R: 5’-ATTCAAAAAGCCTTGAAACAAGCCAAGCTACTCGAG  TAGCTTGGCTTGTTTCAAGGC-3’ |
