# Supplementary material for: SALL4 promotes gastric cancer progression through activating CD44 expression
Source: Oncogenesis. 2016 Nov 7;5(11):e268–. doi: 10.1038/oncsis.2016.69 (PMC5141291; doi:10.1038/oncsis.2016.69)
Supplement: Supplementary Table 2 [file oncsis201669x11.docx]

**Supplementary Table 2.** The sequences of PCR primers for target gene detection

| Gene | Sequence | Size (bp) | T_m_ (^o^C) |
| --- | --- | --- | --- |
| β-actin | F: 5’-CACGAAACTACCTTCAACTCC-3’  R: 5’-CATACTCCTGCTTGCTGATC-3’ | 265 | 60 |
| SALL4 | F: 5’-TCGATGGCCAACTTCCTTC-3’  R: 5’-GAGCGGACTCACACTGGAGA-3’ | 142 | 60 |
| Oct4 | F: 5’-TTGAGGCTCTGCAGCTTAG-3’  R: 5’-GCCGGTTACAGAACCACAC-3’ | 285 | 60 |
| Nanog | F: 5’- CCTGATTCTTCCACCAGTCC-3’  R: 5’-TGCTATTCTTCGGCCAGTTG-3’ | 292 | 60 |
| Sox2 | F: 5’-ACACCAATCCCATCCACACT-3’  R: 5’-GCAAACTTCCTGCAAAGCTC-3’ | 224 | 60 |
| c-Myc | F: 5’-TCAAGAGGCGAACACACAAC-3’  R: 5’-GGCCTTTTCATTGTTTTCCA-3’ | 110 | 60 |
| CD44 | F: 5’- TCACAGGTGGAAGAAGAGAC-3’  R: 5’-CATTGCCACTGTTGATCACT-3’ | 447 | 60 |
| CD44s | F: 5’- GGAGCAGCACTTCAGGAGGTTAC-3’  R: 5’-GGAATGTGTCTTGGTCTCTGGTAGC-3’ | 129 | 60 |
| CD44v6 | F: 5’-CCAGGCAACTCCTAGTAGTACAACG-3’  R: 5’-CGAATGGGAGTCTTCTTTGGGT-3’ | 112 | 60 |
