# Supplementary material for: SALL4 promotes gastric cancer progression through activating CD44 expression
Source: Oncogenesis. 2016 Nov 7;5(11):e268–. doi: 10.1038/oncsis.2016.69 (PMC5141291; doi:10.1038/oncsis.2016.69)
Supplement: Supplementary Table 3 [file oncsis201669x12.docx]

**Supplementary Table 3.** The sequences of primers for luciferase reporter assay and ChIP PCR

| Gene | Sequence | Size (bp) | T_m_ (^o^C) |
| --- | --- | --- | --- |
| CD44-P2 | F: 5’-CGGGGTACCGGCAAGAAGTCCATGCAGAT-3’  R: 5’- CCCAAGCTTATGAGGCTGCCTCGGAAGT-3’ | 1396 | 56 |
| CD44-P3 | F: 5’-CGGGGTACCCTGAGCTGTTCTGCCAAGAAG-3’  R: 5’- CCCAAGCTTATGAGGCTGCCTCGGAAGT-3’ | 646 | 56 |
| CD44 ChIP-PCR | F: 5’-AAAGGCTGAACCCAATG-3’  R: 5’-TGCTCTGCTGAGGCTGTA-3’ | 200 | 52 |
